# The psychosocial barriers to medication adherence of patients with type 2 diabetes: a qualitative study

**DOI:** 10.1186/s13030-020-00202-x

**Published:** 2021-01-18

**Authors:** Firoozeh Mostafavi, Fereshteh Zamani Alavijeh, Arash Salahshouri, Behzad Mahaki

**Affiliations:** 1 Department of Health Education and Promotion, School of Health, Isfahan University of Medical Sciences, Isfahan, Iran; 2 Department of Health Education and Promotion, School of Health, Ahvaz Jundishapur University of Medical Sciences, Ahvaz, 61357 - 15751 Iran; 3 Department of Biostatistics, School of Health, Kermanshah University of Medical Sciences, Kermanshah, Iran

**Keywords:** Medication adherence, Patients, Diabetes mellitus, Type 2, Qualitative research, Iran

## Abstract

**Background:**

The adherence of diabetic patients to their medication regimen is associated with many psychosocial factors that are still unknown. Therefore, the present study aims to identify the psychosocial barriers to medication adherence of patients with type2 diabetes (T2D).

**Methodology:**

This descriptive qualitative study was done in Isfahan, Iran by conducting in-depth unstructured interviews with 23 purposively selected patients with T2D and 10 healthcare providers (HCPs). The participants were interviewed face-to-face between November 2017 and June 2018 at the patient’s home, a Health Care Center, or at the diabetes clinic. Data analysis was performed using MAXQDA-10 software and the conventional content analysis.

**Results:**

The analysis of the data led to six categories of perceived psychosocial barriers: 1) fear, concern and distress, 2) exhaustion and burnout, 3) the children’s issues being the priority, 4) poor financial support, 5) communication challenges, and 6) poor work conditions.

**Conclusions:**

This study identified some of the psychosocial barriers to medication adherence of patients with T2D, which will be of great help to researchers and HCPs in designing and implementing effective interventions to overcome these barriers and change patient self-care behaviors and increase their medication adherence.

## Background

Diabetes Mellitus is a major growing public health problem worldwide [1, 2]. Diabetes is the main cause of blindness, kidney failure, and non-traumatic amputation of the lower limbs [3–5] and also a major risk factor for coronary artery diseases and stroke [6]. Reaching the optimal level of blood sugar and metabolism control to minimize short- and long-term complications is a challenge for patients with T2D and their relatives, and also for Health Care Professionals (HCPs) [7]. In conjunction with lifestyle management [8], medication therapy is also recommended for controlling hyperglycemia [9]. Although adherence to the prescribed medications is a key dimension of the quality of healthcare [10], non-adherence to their medication regimen is commonplace among patients with diabetes [1]. Although the rate of medication adherence varies widely and depends on how it is defined and the study population [11], the rate of adherence to oral hypoglycemic medications among patients with T2D has been reported as 36 to 93% and the rate of adherence to insulin therapy as 63% in some studies [9]. Another study reported the adherence of these patients to insulin therapy as 4.5 to 71% [12]. Despite advances in medical research, the rate of non-adherence has not changed in the last few decades [13]. According to a systematic review in Iran, the rate of Medication Adherence of patients with T2D has been reported to be between 37 and 67% [14]. Previous studies have shown that non-adherence to antidiabetic medications leads to poor blood sugar control and subsequent complications associated with the progress of the disease, hospitalization, premature disability, and patient mortality [15, 16]. Many studies have investigated the factors associated with medication non-adherence, which include medication side-effects, costs of treatment, and poor patient-health service provider interaction [17, 18]. In Iran, research in this area has been mostly quantitative; however, many of the factors affecting the medication adherence of patients are associated with psychosocial factors that remain unknown to date. Therefore, identifying these factors may lead to positive changes in the rate of medication adherence of patients by designing appropriate educational messages and performing the most effective interventions. To explain these unknown factors, quantitative studies alone do not suffice, and qualitative research can better help identify these factors. The present qualitative study was therefore designed and conducted to identify the psychosocial barriers to medication adherence of patients with T2D.

## Materials and methods

### Study design

This was a descriptive qualitative study using conventional content analysis to identify barriers to medication adherence of patients with type2 diabetes. The study was conducted through in-depth unstructured interviews. However, a general interview guide was designed and used by the authors to facilitate the interview process.

### Participants and sampling

Participants were selected using purposeful and/or maximum variation sampling [19] from patients with T2D from patient registration lists in diabetes clinics, healthcare centers, and Health Houses. Inclusion criteria: a heterogeneous group (different ethnic groups, age, sex, marital status, education, and socio-economic status) of patients with T2D; with a history of high or uncontrolled blood sugar (HbA1c ≥ 7% and FBS ≥ 126); a more-than-three-month history of diabetes and taking oral antidiabetic drugs, or taking insulin. To access more comprehensive and in-depth data, other informants were also interviewed, such as HCPs (health professionals, physicians, and primary healthcare workers) with previous experience of patient care and willingness to take part in the program. After 26 interviews the answers were repetitive, but most of the sampled people were illiterate. This led to the selection of seven literate participants and interviews with them. In-depth unstructured interviews were conducted with participants at their home and in their workplace (diabetes clinics, health centers, etc.) until data saturation occurred [4]. Therefore, after interviewing 33 participants, including 23 patients and 10 health care providers, it was assumed that no new data would be added to the analysis. In total, 38 people were invited to participate in the study. Two persons were excluded for not having proper physical and mental conditions to answer the interviewer’s questions and one for sudden death. Also, two persons rejected our offer for interview. Although sessions were organized to answer likely participant’s questions and their possible concerns about recording their voices, some of the participants contacted the researchers directly to receive the information they needed.

### Interview and data collection

The process of sampling continued from November 2017 to July 2018 in the province of Isfahan, Iran. Eventually, 33 people were interviewed face-to-face in Health Care Centers, the patients’ homes, and at the diabetes clinic. Each interview lasted between 20 and 60 min depending on its progress and circumstances and was accompanied with field notes and the recording of non-verbal behaviors and the respondent’s interaction with others. Further details were recorded about the field notes as soon as possible after each interview was over.

In this study, the fourth author (AS) was responsible for conducting the interviews. Twenty-one interviews were conducted at Health Care Centers. Six of the interviews were conducted in the participant’s home, and six at the Diabetes Clinic. Before the interviews, the patients were asked to disclose basic clinical and demographic information, as shown in Table 1.

**Table 1:** Clinical and demographic characteristics

Variable	Diabetes Patients	Health Professionals	Total
	N (%)	N (%)	N (%)
Sex			
Male	10 (43.47)	8 (0.8)	18 (54.54)
Female	13 (56.52)	2 (0.2)	15 (45.45)
Age Group			
20–40	4 (17.39)	9 (0.9)	13 (39.39)
41–60	11 (47.82)	1 (0.1)	12 (36.36)
60 and older	8 (34.78)	0 (0)	8 (24.24)
Marital Status			
Married	23 (100)	6 (0.6)	29 (87.87)
Single	0 (0)	4 (0.4)	4 (12.12)
HbA1c level			
< 7.0%	5 (21.73)		
≥ 7%	18 (78.26)		
Education			
Illiterate	16 (69.56)	0 (0)	16 (48.48)
Junior High School	3 (13.04)	0 (0)	3 (9.09)
High School Diploma	3 (13.04)	0 (0)	3 (9.09)
Associate Degree	1 (4.34)	3 (0.3)	4 (12.12)
Bachelor’s Degree	0 (0)	4 (0.4)	4 (12.12)
Master’s Degree	0 (0)	1 (0.1)	1 (3.03)
PhD	0 (0)	2 (0.2)	2 (6.06)
Residence			
Urban	15 (65.21)	5 (50)	20 (60.60)
Rural	8 (34.78)	5 (50)	13 (39.39)
T2DM History In 1st-Degree Relatives			
Yes	18 (78.26)		
No	5 (21.73)		
Occupational Status			
Employed	4 (17.39)		
Retired	4 (17.39)		
Housewife	10 (43.47)		
Unemployed	5 (21.73)		
Time Since Diagnosis			
< One Year	2 (8.69)		
1 to 5 Years	5 (21.73)		
> 5 Years	16 (69.56)		
Household Income			
Inadequate to Cover Living Expenses	16 (69.56)		
Sufficient to Cover Living Expenses	7 (30.43)		
Complications Of Diabetes^a^			
Yes	8 (34.78)		
No	15 (65.21)		
Physical Activity			
Regular	5 (21.73)		
Irregular	8 (34.78)		
No Physical Activity	10 (43.47)		
Drug Use And Smoking			
Yes	4 (17.39)		
No	19 (82.60)		
Visit By Doctor			
Regular	14 (60.86)		
Irregular	9 (39.13)		
Medications			
Metformin Only	8 (34.78)		
Metformin & Glibenclamide	8 (34.78)		
Metformin & Insulin	7 (30.43)		

^a^ Having at least one microvascular (nephropathy, neuropathy and retinopathy) or cardiovascular (cardiovascular disease, stroke, peripheral artery disease and cerebrovascular disease) complication, hypertension & hyperlipidemia

The interviews were held in the absence of the patient’s family members. The participants were briefed on the study objectives at the beginning of the study and their written consent for recording the interviews was obtained. At the end of each interview, consent was obtained once again in view of the issues discussed in the session. The interviews with patients began with introductory questions aiming to create a friendly atmosphere and the participants were asked to disclose basic demographic information and continued with phrases such as “Please talk about your disease and conditions”, “what do you mean by…”, and “tell me more about”. The next questions were unplanned and were posed based on the interviewer’s experience and discussion of the subject. The interviews were recorded using a tape recorder and were carefully listened to and transcribed verbatim on paper, then input to a computer at the earliest opportunity. After typing the interviews and their review, the authors conducted a second interview with three of the participants in response to the need for further information.

### Data analysis

The typed-up interviews (154 pages) were entered into MAXQDA-10 and were analyzed using conventional content analysis [20].

Each interview text was read several times carefully, and the first-level codes were extracted by breaking down the texts. The initial codes were then categorized according to their similarities and differences, and the second-level codes were then produced by naming each category, repeating the categorization, combining the similar codes, and adding new emerging codes, and the themes thus emerged and were categorized. In fact, the inductive method was used in this study. When all the data were encoded and agreement was reached on the categories, each category was assessed in terms of saturation. Given their previous experiences with qualitative research, the second (FZ) and fourth (AS) authors contributed more to the data analysis and felt that data saturation occurred, as they could no longer discern any new information and additional data collection may no longer generate new understanding.

All the stages of interviewing, typing interviews, and encoding were done in Persian (Native language). In the end, the categorized codes were translated into English. In addition, further discussions with the qualitative research experts enabled AS and FM to be reflexive of assumptions and biases that may have influenced the research process.

### Ethical considerations

Research ethics approval was acquired prior to the commencement of the study from the Research and Technology Deputy of Isfahan University of Medical Sciences (Code of ethics: IR.MUI.REC.1396.3.522 and project No: 396522). The participants were briefed on the study objectives and interview methods and ensured of the confidentiality of their data and their right to withdraw from the study at any time at the beginning of the interviews, and their written consent for participation was also obtained. The interview time and place were arranged with the participants so that they could have sufficient time to participate in the interviews and to share their views.

### Scientific trustworthiness of the results

To evaluate and enhance the scientific trustworthiness and rigor of the results, the criteria recommended by Lincoln and Guba were used, which included credibility, transferability, dependability, and confirmability [21]. To evaluate and enhance the credibility of the findings, we tried to select participants with the maximum diversity of experiences, and sampling continued until data saturation. In order to increase the content validity, member checking was used, so transcribed and encoded data were returned to the participants to confirm and comment. Transferability of data was provided by offering a comprehensive description of the subject, participants, data gathering, and data analysis. Also, to increase the dependability of the research results, an external observer examined the data carefully (External checking). To enhance the confirmability; several research collaborators were given the process of doing the study to confirm the correctness of how to do the research.

## Results

The study subjects included 23 patients (ten men and 13 women) and ten HCPs (eight men and two women; four disease prevention experts, two nutritionists, two primary healthcare workers, and two general practitioners). The data from 33 participants were ultimately analyzed, and their demographic characteristics are presented in (Table 1). Approximately 54.54% of the participants were male and 45.45% were female, 87.87% were married, and 48.48% were illiterate.

The analysis of the data on the determinants of the patients’ adherence to their medication regimen led to the extraction of six main categories, each with a number of subcategories (Table 2). This study investigates the psychosocial barriers and their subcategories to the medication adherence of patients with T2D. Most quotations selected for mention in the article were patterning under different conditions, unless they were only raised by a group of participants.

**Table 2:** The codes, subcategories, and categories of the psychosocial barriers to diabetic patients’ Adherence to their Medication Regimen

Category	Subcategory	Code	
1.Concern, distress and fear	Concern due to the lack of trust	Concern due to the lack of trust in health service providers	The patients’ lack of trust in the physician about the diagnosis of the disease symptoms and complications
		Concern due to the lack of trust in the medications	Concern about the effect of the medications
			Concern about the medication side-effects
	Psychological distress due to the calamities of life	Distress about the death of a family member (sister, brother)	
		Distress about relatives’ illness and death	
	Concern about being a burden		
	Concern about being neglected by the family members		
	Concern about financial problems	Neglecting medication adherence due to financial pressures	
	Fearing the community’s bad reactions to the disease	Fearing the stigma caused by having diabetes	The disease being considered hereditary in the family by others
		Fearing people restricting their relationships with diabetic patients	The disease being considered contagious by others
	Fearing the experience of hypoglycemia		
2.Feeling exhausted and burnt out	Getting tired of the prolonged period of treatment		
	Feeling exhausted as a result of the calamities of life		
3.Prioritizing the children’s issues	Giving priority to the children’s needs		
	Giving priority to the children’s disease		
4. Poor financial support	No financial support provided by the family for the purchase of the medications		
5. Communication challenges	Advertisements encouraging non-adherence to a medication regimen	Advertisements and encouragements about the use of herbal medicines by other patients and relatives	
		Advertisements and encouragements about the benefits of vegetarian and raw food diets by other patients	
	Poor communication processes	Inadequate physician consultation about the medications	
		Poor physician-patient interactions	
6.Poor work conditions	Forgetting to take the medications as a result of being too busy		
	Not taking the medications on time due to long work hours		

### Fear, concern, and distress

According to the results obtained, a large number of the participants considered fear, concern and distress as the main reasons for the non-adherence of patients to their medication regimen. Based on the analysis of the data, the participants proposed seven reasons for non-adherence to medication regimen as a result of fear, concern and distress barriers, including:

#### Concern due to distrust

According to the participants, the patient’s concern due to distrust in the scientific capability of the treatment supervisors was a major factor in the therapist-patient relationship that greatly affected the patient’s adherence to treatment and therefore recovery, such that the patient’s loss of trust in the physician or the treatment supervisor diminished his strictness in adherence to the prescribed medication regimen. One of the patients said about this lack of trust: “One of the reasons for not using the medications prescribed by my doctor was that I did not trust his scientific prowess and was constantly worried about his medical knowledge not being good enough for treating me” (A 32-year-old male patient with diabetes).

Another participant remarked: “After nearly two months, my doctor didn’t even realize that I had a blood sugar problem, so I couldn’t trust him any longer” (A 32-year-old male patient with diabetes).

The participants also stated that the lack of trust in the effect of anti-diabetes drugs concerned them and made them doubtful about whether or not to use these medications. For instance, a 64-year-old male patient with diabetes said: “I didn’t take my medications at first because I was worried they wouldn’t really help control my blood sugar”.

Participants’ experiences revealed that they feared the side-effects of the medications so much that some of them believed their physical ailments were caused by diabetes medications, and these concerns about medication had largely prevented their adherence to the recommended medication regimen.

A 50-year-old male patient with diabetes said: “Diabetes medications have side-effects for the kidney, liver, etc., so I had the right to be concerned, and I may have refrained from taking my medications just to reduce these concerns”.

One of the treatment supervisors said: “Some patients blame these medications even for their knee pain and wish to discontinue or change their medications”.

#### Concerns and mental anguish following a calamity of life

Another factor affecting the non-adherence of diabetic patients to their medication regimens was the mental distresses caused by a calamity of life. The loss of family members and relatives and bereavement were among the highly distressing life events that led to the loss of desire, motivation, and sense of ability to adhere to a medication regimen. One of the participants said: “Sometimes I get so sad about my brother’s death that I cry. I get so down that I may not take my medications even for a couple of days” (A 44-year-old female patient with diabetes).

The concern and distress following an illness and problems of family members or others was another psychological factor that was discussed by the patients as a deterrent to medication adherence. One of the participants explained: “My niece’s cancer has affected us all and I’m in such psychological turmoil that can’t even think about my diabetes and its treatment” (A 35-year-old female patient with diabetes).

#### Concern about being a burden

Another psychological factor that created a barrier to the medication adherence for patients with T2D was the feeling of being a burden. One of the patients said: “I felt like a burden to my family and had no reason to live, so why bother taking the medications?” (A 62-year-old male patient with diabetes).

#### Concern about being neglected by family members

According to the participants, some patients were neglected by their family members and did not have much reason to continue their treatment or go on living.

One of the patients said: “Neither my son nor his wife paid any attention to me, my illness didn’t matter at all to them. It was as if I was one too many. So, I had no reason to live or bother taking my medications” (A 62-year-old male patient with diabetes).

One of the treatment supervisors said: “The patients’ problems and their childrens’ neglect and lack of affection are reason enough to have no motivation to live and take their treatment seriously” (A 35-year-old man, prevention expert).

#### Concern about financial problems

The patients said that financial problems caused them distress and psychological pressure and created a barrier to their medication adherence.

A 44-year-old female patient with diabetes said: “Sometimes, the financial pressures in life are so massive that I can’t be bothered with my disease or medications”.

#### Fearing society’s bad reactions to the disease

This category consisted of two subcategories, including fearing the stigma caused by diabetes and fearing people restricting their relationships with diabetic patients. Fearing the disease being considered hereditary in the patient’s family and fearing the disease being considered contagious by others due to the lack of public awareness about the disease made some of the patients hide their disease from others as much as possible, at times even making them avoid taking their medications or having it injected in the presence of others and occasionally making them not allow their treatment supervisor to follow up on their condition. A 55-year-old female patient with diabetes said: “During my one year of illness, I didn’t inject insulin until I was alone. When we had a guest at home, I wouldn’t inject my insulin at all, fearing that they might learn about my illness”.

Another patient said: “My son and his wife have been living with us for two years now, but I never used my medications whenever his wife was home because I didn’t want her to know that we have this disease in our family” (A 54 year-old female patient with diabetes).

One of the patients said to his treatment supervisors: “I would be embarrassed if others found out that I have diabetes and stopped hanging out with me out of fear, so I don’t want you to come here to supervise my medication use any longer” (A 55-year-old male patient with diabetes).

#### Fearing the experience of hypoglycemia

The analysis of the results showed that fearing the experience of hypoglycemia has a major role in the non-adherence of patients to their medication regimen and thereby their disease control. “Injecting insulin made me lethargic and weak. Because my glucose level dropped and I could not stand on my feet, I didn’t inject my insulin many times out of this fear” (A 60-year-old female patient with diabetes).

### Exhaustion and burnout

Patients becoming tired of continuing their long-term medication therapy and feeling exhausted with life and burnt out as a result of dealing with problems comprised another psychological barrier to adherence to medication therapy.

A 44-year-old female patient with diabetes said: “I used to ask myself how much longer I would have to take these medications? I’d gotten tired of it and wouldn’t take them any more”.

One of the supervisors discussed the patients getting tired of their life and its hardships and the effect this exhaustion had on their treatment: “Diabetic patients in this region have suffered a lot and somehow consider death as a relief, so they have no desire to take their medications” (A 35-year-old man, disease prevention expert).

### Prioritizing the children’s issues

Some of the patients gave priority to their children’s needs and illness and cared about their family’s circumstances more than they cared about their own treatment. “I didn’t want my kids to be envious of the other kids’ clothes, so, I thought more about my children rather than worrying about buying my medications” (A 44-year-old female patient with diabetes).

Another patient commented: “I haven’t been spending my money on my medications and tests for a while and spend it entirely on my son’s treatment, because my kid’s disease is more important to me” (A 42-year-old female patient with diabetes).

### Poor financial support

Some of the patients complained about the lack of financial support from their families for their treatment: “My children were in a bad financial state and couldn’t help me get my medications” (A 51-year-old female patient with diabetes).

“Unfortunately, my family has their own problems and can’t support me to get my medications” (A 54-year-old female patient with diabetes).

### Communication challenges

In the participants’ experience, communication challenges in the adherence of diabetic patients to their medication regimen fell into two subcategories:

#### Advertisements encouraging refraining from adherence to medication regimens

These advertisements are one of the barriers discussed by many of the participants, and other diabetic patients recommendation of herbal medicines and exaggerating and advertising their effects were a reason for the patients’ inclination toward using herbal medicines instead of antidiabetic medications. “Some women neighbors told me of herbal medicines such as the common yarrow and said that they’re good for blood sugar, so I’ve been taking those instead of my regular medications” (A 51-year-old female patient with diabetes).

Another patient said: “I used Hawthorn leaves or other plants recommended by others instead of my medications” (A 66-year-old female patient with diabetes).

A 64-year-old female patient said: “One of the patients told me that she had used the same herbal medicines and that her sugar had dropped by 400 units, so based my decisions on what she told me, I only use herbal medicines”.

According to the participants, other patients advertising the effect of vegetarian diets and raw food diets on blood sugar encouraged them to become vegetarians, to follow raw food diets, and not use their diabetes medications.

“When the patient said that her blood sugar had dropped by eating only raw foods and following a vegetarian diet, I got tempted to become a vegetarian and eat raw foods instead of taking my antidiabetic medications” (A 32-year-old male patient with diabetes).

#### Poor communication processes

Communication barriers were divided into two subcategories, including inadequate physician consultation and poor physician-patient interaction.

Some of the patients complained about the poor information support and inadequate counseling they received from health service providers. A 54-year-old male patient said: “The physicians and treatment supervisors often spend little time giving us information about our medications, their dosages, and possible side-effects. We need more information …. But the physicians have no time”.

A 55-year-old female diabetic woman discussed the role of poor communication with the treatment supervisors in the patients’ medication adherence: “To make me take my medications, the doctor suddenly started talking about my death just out of the blue. He treated me as if my life or death didn’t matter to him at all. This behavior discouraged me and I never visited him again or used the medications”.

### Poor work conditions

Poor work conditions comprised another social barrier to the patients’ adherence to the medication regimen. The analysis of the results showed that the patients’ inappropriate work conditions and preoccupations made them forget to use their medications on time. A 50-year-old female patient said: “I used to forget to take my pills before lunch because I was so busy”.

Some of the patients said that they had difficulty taking their medications on time because they had to stay at work until late. “It was difficult for me to take my medications regularly because I had to be at work until 3.45. That is why I couldn’t take my medications on time” (A 45-year-old male patient with diabetes).

## Discussion

Concern, distress and fear, exhaustion and burnout, giving priority to the children’s needs, poor financial support, communication challenges, and poor work conditions were among the identified barriers in this study. Although some of these barriers may have been discussed in other studies, the present findings provide not only a different combination and categorization of them, but also pave the way for further studies and interventions by defining or re-defining certain concepts. This section aims to compare these results with the results of other studies and explain the barriers and their similarities and differences.

The findings revealed “concern, distress and fear” as the main barriers to medication adherence, and concern due to the lack of trust in the effect of medications and their side-effects was discussed by most patients as a major barrier. The results of a study conducted in China by Shen et al. showed that patients have a greater tendency to use western medicines to control their blood sugar, which is largely due to their trust in the greater effectiveness of western medicines and concerns about the ineffectiveness of their local counterparts in the control of blood sugar [22]. Because, in the view of the patients, traditional Chinese medicines are usually made from plants and other natural sources, they are more compatible with the human body than western medicines and cause less harm. In some cases, concern about the harms of western medicines for the body make the patients turn to Chinese herbal medicines [22]. The results of a study conducted in Palestine showed that there is a significant relation between concerns about medication use and the medication adherence of patients with T2D; when patients have adequate information about the importance of the medications and their therapeutic effects and possible side-effects and other related issues, they develop a greater acceptability for them [23].

“Concern about the lack of trust in the therapists’ scientific knowledge and their medical recommendations” was one of the barriers discussed by some of the patients. This concern can be largely attributed to poor physician-patient interactions, which was also discussed as a major barrier. If proper communication is established between the therapists and the patients and proper counseling sessions are held for them about the disease and its medications and their side-effects, and if the patients’ suggestions about the treatment are accepted, their trust will increase in the therapists and their concerns due to distrust in the medications will also be greatly abated. In line with the present findings, the results of a study conducted on adults with T2D showed that poor physician-patient communication is a major barrier to disease management; the patients examined in the study were unhappy that they could not share their views on their own treatment and felt that the physicians had a father-like approach to their mutual communication [24]. In the present study, poor communication was not only discussed as a cause of this concern, but the patients’ lack of trust in the physicians’ capability also comprised an important dimension of this concern – the latter of which has less been addressed in previous studies.

Adherence to treatment regimen is one of the key dimensions in the self-care of patients with T2D that can be affected by “psychological distress” [25, 26]. The present study identified a new categorization for the dimensions of this effective factor in the study population. The results showed that, in some cases, diabetic patients experience psychological distress due to the death or incurable illness of family members or relatives and depression following bereavement and might then less adhere to their medication regimen. Although no similar studies were found with which to compare these findings, it is evident that the intense affection Iranians tend to have for each other makes coming to terms with the calamities of life difficult for most of them and may end in their lack of motivation to pursue their treatment. These situations need to be identified and educational interventions need to be planned for reducing their effects on treatment processes.

Concern about being a burden and being neglected by family members was another psychological barrier to medication adherence. Most patients with T2D are older adults [27]. Old age is a critical period of life for everyone, because an old adult loses his job, perceives his children as separated from him, and feels lonely, in a sense. In addition, his physical and mental capabilities diminish and he might view himself as a burden to the community and the family; it is therefore possible for him to consider certain actions by his family members as signs of neglect. In such cases, the patient loses his motivation for continuing the treatment and may consider ending his life and see his death as a relief. It is the duty of both healthcare workers and family members and relatives to pay significant attention to the psychological and emotional support of their older patients with diabetes.

An important barrier to the medication adherence of diabetic patients was “Fearing the community’s bad reactions to the disease”, with most of the patients revealing that they concealed their disease as much as possible to avoid the stigma associated with it, avoided taking their medications in the presence of others, and did not even allow treatment supervisors to monitor their treatment and care. In a study conducted by Liu et al., the majority of patients with T2D (52%) reported that there is a certain stigma associated with diabetes [28]. The results obtained by Gredig et al. in Switzerland showed that patients who reported higher levels of perceived stigma associated with diabetes had a greater anxiety, showed more symptoms of depression [29], and reported that their disease had negative effects on their relationships with their family, friends, and peers [30]. The participants of a study conducted by Purnel et al. expressed concerns about their local cultural norms and the social stigma associated with self-care behaviors, and the fear of being labeled as addicts in the community made them feel embarrassed about injecting insulin to themselves in the presence of others [24]. Two of our findings, namely, participants’ fear of the disease being considered hereditary by others and fearing that people may end their relationship with them out of the fear of diabetes being contagious, indicate the innovative aspect of the present study, in other words, they demonstrate cultural differences between Iran and other countries. According to some other studies, ‘weak’ [31], ‘fat’, ‘lazy’, ‘voracious’, and ‘greedy’ are some other labels attached to diabetic patients [32], and some patients may conceal their disease from others to avoid these labels. The development of programs and strategies to protect diabetic patients against stigma should be on the top agenda of HCPs, so that significant progress can be observed in society’s acceptance and support of these patients.

“Fearing the experience of hypoglycemia” was extracted as one of the factors preventing adherence to treatment. The incidence of hypoglycemia is always possible for any diabetic patient receiving medication therapy. Excessive insulin injection, excessive outdoor activity or missing a meal can cause this complication, and the fear of the incidence is ever-present in the life of a diabetic patient. Although a certain level of fear is necessary for warning and informing about threats, in this scenario, excessive fear is dangerous and may prevent the patient’s adherence to the medication regimen. Because some diabetic patients suffer from excessive fear, interventions to abate their fear of hypoglycemia and its adverse effects are necessary [33]. In the present study, fear of hypoglycemia had occurred following insulin injection. In line with these findings, several other studies on the risk/incidence of hypoglycemia as a major predictor of its fear have shown that intensified insulin therapy can exacerbate the fear of hypoglycemia [33, 34].

“Feeling exhausted and burnt out” is a psychological disorder in human societies that can be generated as a result of stressful life events, especially chronic diseases [35]. Following the prolonged process of treatment due to the chronic nature of the disease, and also the experience of hardships in life, patients with T2D may become exhausted with their life and refrain from pursuing their treatment and self-care. Health service providers and treatment supervisors who are in close contact with chronic patients should collaborate with the patients’ family members to identify the reasons for their exhaustion and adopt strategies to help these patients. Evidence is indicative of few psychological interventions carried out in healthcare centers for diabetic patients.

Another barrier to adherence to the medication regimen, especially for married patients, was “giving priority to the children’s needs and issues over one’s own treatment”. Iranian families tend to prioritize their children’s needs and expectations over all other family issues; for parents in these families resolving the children’s problems takes precedence even over their own treatment.

The majority of the participants in the present study referred to the role of “poor support” in their adherence to treatment. The patients regarded support from their family, especially financial support, essential to their empowerment. In line with the present findings, Shen et al. also emphasized the effect of family support (especially financial support for medical expenses) on the self-management behaviors of diabetic patients [22]. In the present study, the negative effects of poor financial support by the family on patient empowerment to adhere to treatment was highlighted because most patients were financially dependent on their families, so that, even when they were aware of the importance of treatment, financial pressures and the heavy costs of the medications affected the patient’s decision to adhere to their medication regimen.

The “advertisements and encouragements of other patients and relatives” to refrain from medication adherence was another factor that was proposed by the participants as a major barrier to adherence to medication regimen. Other patients’ advertising of the effect of herbal medicines and raw food diets and their recommendation of these alternative regimens to other patients (although aiming to control the disease) made some patients refrain from using their antidiabetic medications. The results of a study conducted in East Uganda showed that traditional medicine and its high acceptability in the community was the reason for patients’ willingness to switch to traditional medicine. The study also noted the effect of family, friends, and traditional healers on the acceptance of herbal medicines [36]. In the present study, the results showed the patients’ high susceptibility to the advertisements and views dominating the community. It is therefore necessary to use this strategy for the patients’ adherence to antidiabetic medication regimens.

Another one of the barriers to medication adherence proposed by the participants was “poor work conditions”. The participants sometimes forgot to take their medications as a result of being too busy. In addition, long work hours meant that the patients were unable to take the mid-day dose of their medications. Although the researchers found no similar studies with which to compare these results, given that most patients with T2D are still of working age, this issue needs to be further studied so that effective work-appropriate strategies can be adopted to increase the medication adherence of working patients.

### Strengths and limitations of this study

To increase the credibility of this study, we used both patients and health care providers to answer the research questions from different perspectives.

- In this study, we specifically sought to identify the psychosocial barriers to medication adherence, and this is something that is scarcely addressed in other studies (particularly psychological factors).
- The limitations of this study include the unwillingness of some of the patients (because of fearing the stigma caused by having diabetes) and diabetes treatment supervisors to cooperate with the researchers; however, this limitation was somewhat overcome by briefing the participants on the study objectives and assuring them of their anonymity throughout the research.
- The limitation of funding and timing restricted our ability to conduct a more complete review study; therefore, results of this study are not truly representative of the entire population.

## Conclusion

This study identified some of the psychosocial barriers to medication adherence of patients with T2D that can help researchers and health service providers design and implement effective interventions to overcome these barriers and change patients’ self-care behaviors, thus increasing their medication adherence. Given that psychological problems have a major role in the process of treatment for T2D, it is recommended that patients with T2D be screened for psychological problems and that those eligible for counseling be referred to experts in the mental health departments of healthcare centers in conjunction with their medical treatment. Educational interventions should also be implemented to increase the patients’ and the community’s awareness about diabetes facts and to reduce their sensitivity to the stigma of this disease.

## Data Availability

The interview data that supports the findings of this study are available, but our consent form did not allow for participant interviews to be shared beyond the study team. However, some of the datasets are available from the corresponding author on reasonable request.

## Abbreviations

T2D: Type 2 diabetes
HbA1c: Haemoglobin A1c or glycated haemoglobin
MAXQDA: Qualitative data analysis software and Mac OS X
HCPs: Healthcare providers
